# Study on Mechanical Properties of Multi-Cavity Steel-Concrete Composite Beam

**DOI:** 10.3390/ma15144882

**Published:** 2022-07-13

**Authors:** Chunbao Li, Hui Cao, Di Guan, Shen Li, Xukai Wang, Valentina Y. Soloveva, Hojiboev Dalerjon, Zhiguang Fan, Pengju Qin, Xiaohui Liu

**Affiliations:** 1 College of Pipeline and Civil Engineering, China University of Petroleum (East China), Qingdao 266580, China; z20060072@s.upc.edu.cn (H.C.); z20060042@s.upc.edu.cn (X.W.); 2 Construction Project Management Branch of China National Petroleum Pipeline Network Group Co., Ltd., Langfang 065001, China; guandi@pipechina.com.cn (D.G.); lishen@pipechina.com.cn (S.L.); 3 Emperor Alexander I St. Petersburg State Transport University, 190031 St. Petersburg, Russia; soloviova-pgups@mail.ru; 4 Mining-Metallurgical Institute of Tajikistan, Buston 735730, Tajikistan; gmit_tajikistan@mail.ru; 5 Henan Huatai New Material Technology Corp., Ltd., Nanyang 473000, China; jennifer@gmail.com; 6 College of Civil Engineering, Taiyuan University of Technology, Taiyuan 030024, China; qinpengju@tyut.edu.cn; 7 Qingdao Urban Development Group Co., Ltd., Qingdao 266061, China; liuxiaohui2014upc@163.com

**Keywords:** multi-cavity steel plate, composite beam, mechanical properties, ANSYS

## Abstract

This paper proposes a new form of composite beam: a multi-cavity steel-concrete composite beam. This composite beam uses internal perforated steel plate to connect the concrete with the steel structure, and shear connectors are no longer required, which is more suitable for industrial production. The mechanical properties of a multi-cavity steel-concrete composite beam in industrial applications are studied to avoid failures. In this paper, two multi-cavity steel-concrete composite beams with a size of 2500 mm × 200 mm × 300 mm were prepared, in which the angle of internal porous steel plate was set as 60° and 75°, respectively. A full-scale static load test was conducted on the beams to research its deformation and failure modes. The finite element software ANSYS was used to perform finite element modeling of multi-cavity steel-concrete composite beams and to analyze the influence of concrete strength, steel strength, porosity, and the angle of internal porous steel plate on the mechanical properties of composite beams. The results are as follows: before the composite beam reaches its serviceability limit state, its deformation basically shows a linear change; with the increase of load, the plastic deformation is gradually obvious, which can still provide a certain bearing capacity in the failure stage; the bearing capacity of the composite beam is positively correlated with the strength of concrete and steel, while negatively correlated with the porosity and the angle of internal porous steel plate; composite beams have large bearing capacity, good ductility and integrity.

## 1. Introduction

With the development of the industrialization of buildings, composite structures have been widely applied in construction engineering [1,2,3]. Steel-concrete composite structure is currently the most common type of composite structure, which combines the advantages of steel structure and concrete structure to better meet market demands [4,5,6]. Beams are an important part of the engineering structure [7,8]. Compared with the traditional reinforced concrete beam, the steel-concrete composite beam has the obvious advantages of light weight, high bearing capacity and high stiffness, which greatly reduces the generation of concrete waste [9,10]. As an important horizontal bearing component, this kind of structure can be widely used in industrial buildings, underground structures, high-rise buildings and bridge structures [11,12,13]. Among them, steel-concrete beams in industrial buildings are the most widely used, and their forms mainly include concrete-cladding composite beams, steel-exposed composite box beams and steel-exposed composite T beams [14,15,16,17]. Although there are many types of composite beams, their application is limited due to actual conditions and construction costs.

To make the steel-concrete composite beam better used in engineering practice, a lot of research on its mechanical properties have been conducted. Barnard et al. [18] proposed a method to predict the ultimate flexural capacity of steel-concrete composite beams based on the study of the stress-deformation curve of concrete. Subsequently, Johnson et al. [19] adopted the limit analysis method to study the ultimate flexural bearing capacity of continuous steel-concrete composite beams. Dai et al. [20] studied the influence of stud shear connectors on the performance of composite beams. The results showed that the increase of concrete strength has a great influence on the increase of stud bearing capacity. Hou et al. [21] further analyzed the influence of the shear stiffness of the stud as the connector on the free vibration characteristics of the steel-concrete composite beam. The analysis showed that the influence of the stiffness of the stud shear connector should be considered in the dynamic performance analysis and impact coefficient calculation of the composite beam.

To make better use of the performance of the two materials in steel-concrete composite beams, many new types of steel-concrete composite beams have been developed in recent years. Chen et al. [22] proposed a steel-concrete composite beam, the beam ribs of which were U-shaped steel sections, while it and the ribs and upper flanges were poured with concrete and the steel plate and concrete were connected by shear connectors. The mechanical properties of the specimens were analyzed and it was concluded that the shear connectors between the concrete part and the outer plate steel beam could achieve effective synergy, and therefore the specimens had good overall performance. Yang [23] proposed a type of composite beam, the main body of which was H-shaped steel, and it was formed by welding tie bars inside the web cavity and filling concrete. Experiments proved that the flexural performance of this composite beam was greatly improved.

At present, steel-concrete composite beams are mostly formed by connecting steel and concrete with connectors, which weakens the integrity of the connection between steel structure and concrete, and the construction process is complicated. To further enhance the integrity between steel structure and concrete, simplify the construction process, and further improve the application of steel-concrete composite beams in industrial factory buildings, based on the previous research, this paper proposes a new type of multi-cavity steel-concrete composite beam component. The multi-cavity steel-concrete composite beam is made of thin steel plates welded as the composite beam shell, internal porous steel plate welded with a certain angle, and the filling concrete, so that the integrity of the connection between concrete and steel plate is strengthened without the requirement of shear connector, and the construction process is simplified. Combined with previous research methods, this paper investigated the mechanical properties of multi-cavity steel-concrete composite beams by means of full-scale static load test and finite element analysis. Firstly, the structural type of composite beam was described in detail. Two multi-cavity steel-concrete composite beam specimens were prepared, and the angle of internal porous steel plates was set as 60° and 75°. The full-scale static load test and relevant material tests were carried out at room temperature, and the deformation and failure characteristics were observed. Then the data were collected, and the load-deflection curve and load-strain curve were obtained and analyzed. Secondly, ANSYS finite element modeling calculation was used to compare the simulation results and experimental results to verify the feasibility of the model; Thirdly, the optimized model was used to conduct factor analysis on the composite beam and its multi-cavity steel frame to study the influence of the concrete strength, steel strength, the porosity of the internal steel plate and the angle of porous steel plate on the normal bearing capacity of the composite beam.

## 2. Structure Type

**Figure 1:**
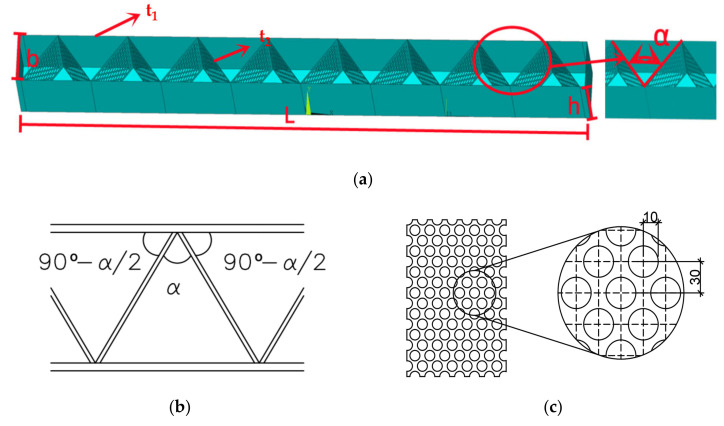
Multi-cavity steel plate: (**a**) Schematic diagram of multi-cavity steel plate frame with partial details; (**b**) Schematic layout of porous steel plate; (**c**) Schematic diagram of pore distribution.

The multi-cavity steel-concrete composite beam is composed of a multi-cavity steel plate framework and concrete. A schematic diagram of the multi-cavity steel plate framework with partial details is shown in Figure 1a. As shown in Figure 1a, the multi-cavity steel frame is welded by the outer steel plate and the porous steel plates with an angle α to each other. The detailed distribution diagrams of the layout and grid of the porous steel plates of the multi-cavity steel plate framework are shown in Figure 1b,c. Because the steel plate has holes, it can strengthen the overall bonding performance of the concrete and the multi-cavity steel plate framework and improve the integrity of the composite beam [24]. Compared with a traditional concrete beam, the multi-cavity steel-concrete composite beam eliminates the process of tying steel bars and supporting templates, improves the construction speed and reduces costs. This composite beam is feasible for industrial production with standard specifications and attractive appearance.

## 3. Test Scheme of Multi-Cavity Steel-Concrete Composite Beam

### 3.1. Design and Production of Test Specimen

To research the bearing capacity and deformation characteristics of the multi-cavity steel-concrete composite beam, two multi-cavity steel-concrete composite beams were prepared and tested.

**Figure 2:**
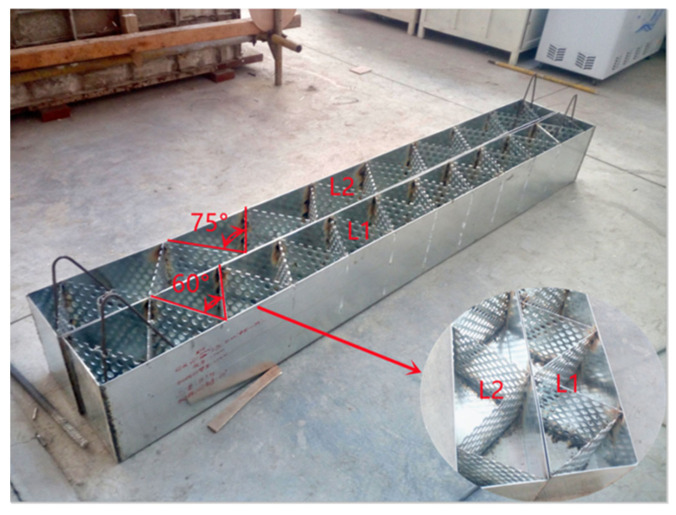
Detailed of multi-cavity steel plate framework and parts.

The design size of the two composite beams (b×h×L) was 200×300×2500 mm, and the angle of the internal steel plate of the composite beam was 60° and 75°, respectively. The design thickness of the outer steel plate (t_2_) in the multi-cavity steel plate was 1.7 mm, and the design thickness of the internal porous steel plate (t_1_) was 1 mm. The steel type used for the multi-cavity steel plate was Q235, and the self-compacting concrete was produced with a compressive strength of C30. Detailed information of the porous steel plate including the pore diameter (r) and center distance (d) is listed in Table 1. Detailed information of the porous steel plate including the pore diameter (r) and center distance (d) is listed in Table 1. A detailed diagram of the multi-cavity steel plate framework and parts used is shown in Figure 2.

### 3.2. Material Test

The steel used in the mechanical property test of steel materials was the same as the outer steel plate and the internal porous steel plate in the composite beam, both of which were Q235 steel. According to Chinese code GB/T 228-2002 [25], three specimens were prepared for the 1.0 mm and 1.7 mm steel plates used in the test, and tensile test [26] was conducted at room temperature. The obtained yield strength was 236.5 MPa and 236.4 MPa, respectively. The concrete used in this test was self-compacting concrete, which required no vibration in pouring process. The concrete used in the mechanical property test and the concrete for the composite beam were from the same batch, and the strength was C30. Nine standard test blocks of concrete cubes of 150×150×150 mm were prepared according to Chinese code GB50010-2010 [27]. The average compressive strength of cubes was 29.30 MPa, which reached 97.68% of the design grade standard value. The elastic modulus was 27.67 GPa. In this test, the multi-cavity steel plate framework was mainly composed of two parts: the outer steel plate and the inner porous steel plate, which were welded together. The welds used in the specimens all complied with the welding standards in Chinese code GB 50017-2003 [28].

### 3.3. Test Measurement

**Figure 3:**
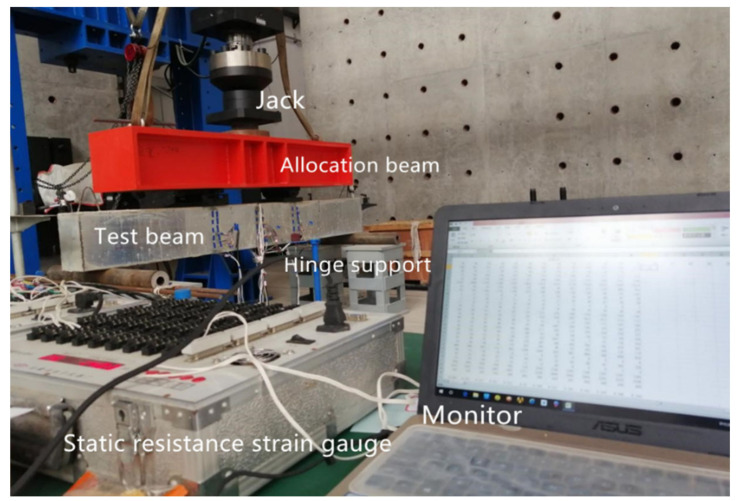
Diagram of the static load test test device.

**Figure 4:**
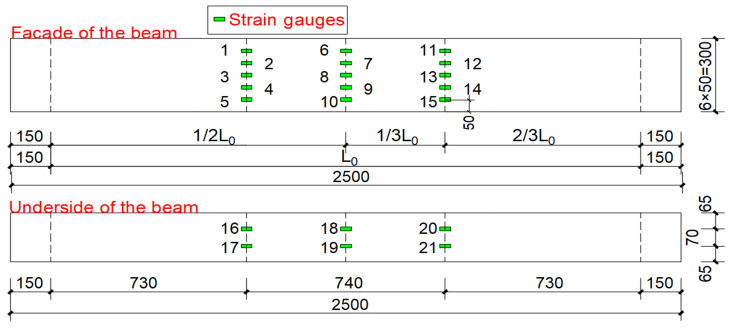
The layout of strain measurement points.

Figure 3 is a diagram of the static load test device. The round steel tube is the support of the composite beam, making it simply supported. Under the round steel tube is a steel pier with great stiffness used to raise the specimen to the appropriate position and prevent it from touching the ground when the deformation is too large during the experiment, which will affect the test results. The net span of the specimen is $L_{0}$, and $L_{0}$ can be calculated as the length minus the support distance. Strain gauges were arranged on the outer steel plate at 1/2 and 1/3 of the net span of the specimen to measure the strain variation value of the specimen. The arrangement of strain gauge is shown in Figure 4.

### 3.4. Loading Scheme

**Figure 5:**
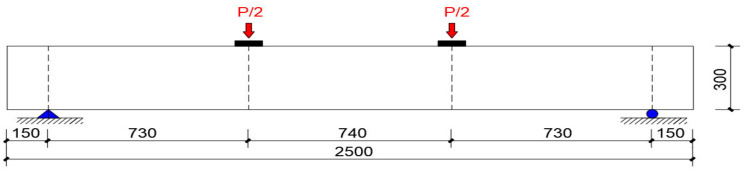
Schematic diagram of loading scheme.

The press machine was equipped with the JAW-2000K multi-channel electrohydraulic servo loading system, and the maximum vertical loading capacity was 2000 kN. In the composite beam specimen, 150 mm inward from both ends were taken as simple fulcrums, and the network span between the two fulcrums was the research object. The vertical concentrated load was applied at the two three-points of the net span of the test specimens. The total load was equally divided into two parts by the sub-load beam and the force transmission abutment placed between it and the composite beam specimen and converted into surface load and applied to the upper surface of the composite beam. The schematic diagram of the loading scheme is shown in Figure 5. Before loading, the position of the composite beam test specimen was adjusted to keep level, followed by pre-compression until the upper bearing component of the test specimen closely fitted the press. When the test preload requirements were met, the preload values of L1 and L2 were 4.7 kN and 6.0 kN, respectively. Then, the load was graded by sections and entered the formal test stage. For the first stage: about 5 grades of load, 1 kN for each grade. The contact surface changes and micro-deformation of composite beam specimens were observed at the initial stage of loading to further determine the compactness of the contact surface. For the second stage: about 5 grades of load, 2 kN for each stage. This stage was the transition period for formal loading. For the third stage: each stage was loaded with 4 kN to the normal limit (i.e., the deflection limit of the flexural component (L⁄250=8.8 mm), and then several levels of load were loaded to observe the deformation characteristics of the composite beam specimens before and after the bearing capacity limit. For the fourth stage: 8 kN for each grade until the specimens were crushed, and the ultimate bearing capacity and failure mode were observed. See Table 2 for the loading process of each section of composite beam specimen L1 and L2.

## 4. Test Results and Analysis

### 4.1. Test Phenomenon and Failure Mode

**Figure 6:**
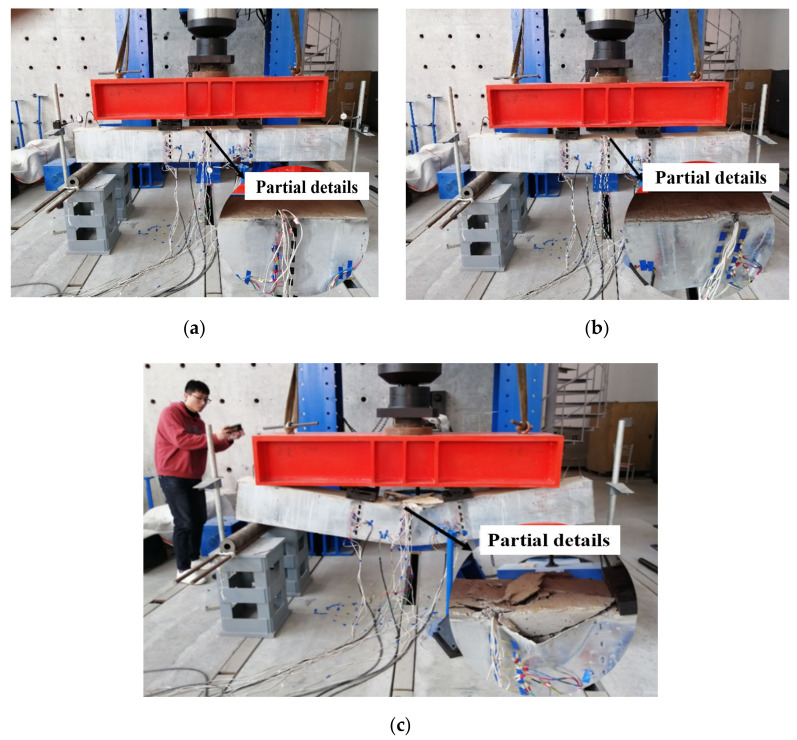
Destruction diagram of composite beam specimen: (**a**) Close attachment stage; (**b**) Micro-detachment stage; (**c**) Failure stage.

According to Chinese code GB 50010-2010 [26], the deflection limit value of this composite beam is L⁄250=8.8 mm. Based on the deflection changes and failure of two composite beams, the test process of this paper can be roughly divided into three stages. Since the failure phenomena of the two composite beams are similar in the test, the picture of L1 beam with 60° was adopted as an example to show the test process. The first stage was the close-fitting working stage, in which the mid-span deflection of the composite beam specimen didn’t change significantly, and the outer steel plate was always closely attached to the internal concrete body, as shown in Figure 6a. The second stage was the micro-disengagement expansion stage. This stage began with the occurrence of micro-disengagement and ended with that the mid-span deflection reached the deflection value limited by the limit state of normal use. The detachment between mid-span steel plate and concrete was obvious, but the steel plate only bulged outwards without large deformation, as shown in Figure 6b. The third stage was the failure stage. This stage began with the point where the composite beam specimen reached the limited deflection value and ended when the specimen was crushed. The concrete body in the span of the specimen was crushed and the side steel plate had a large buckling deformation. The bottom steel plate was obviously stretched, but not broken. At this time, the composite beam specimen was completely destroyed, as shown in Figure 6c. The failure phenomenon in the third phase resulted from the mechanical properties of the composite beam of the lower part in tension and the upper part in compression. The lower part of the multi-cavity steel-concrete composite beams was steel plates with good tensile performance; when the steel plate exceeded the limit state of normal use, it didn’t break, and the upper concrete was only partially damaged under pressure. Although the steel plate had a large buckling deformation, the concrete and steel plate still had a large adhesion, showing a better integrity.

### 4.2. Load-Deflection Curve

**Figure 7:**
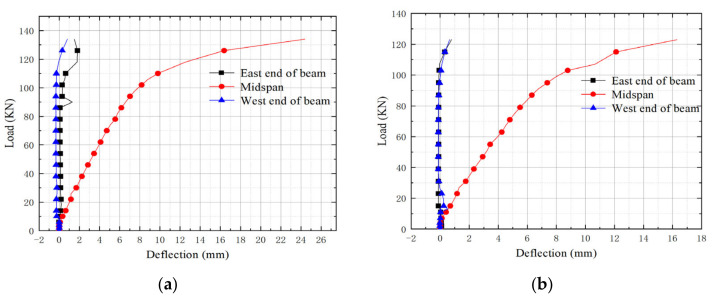

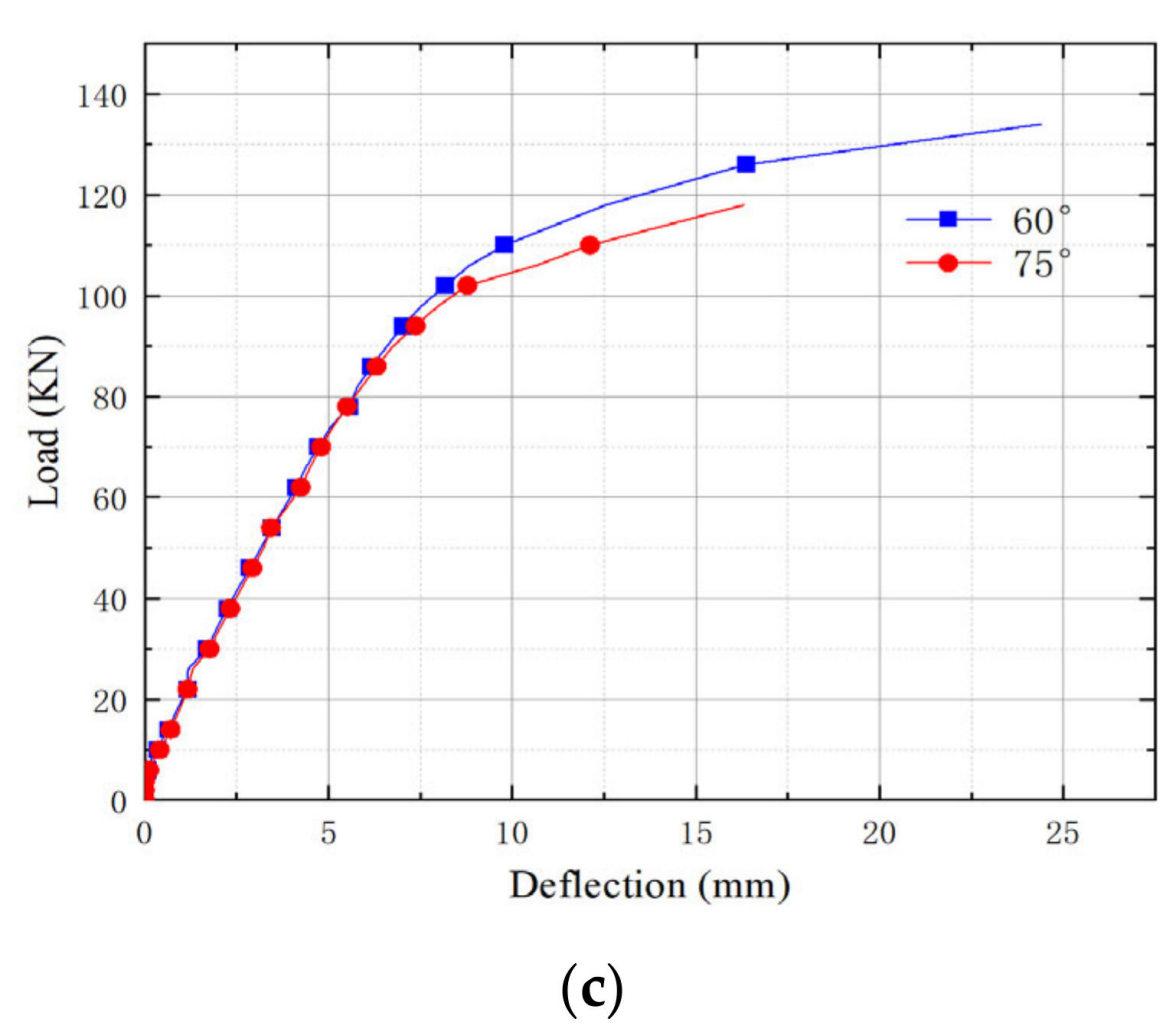
Composite beam load-deflection curve: (**a**) L1-60°composite beam; (**b**) L2-75° composite beam; (**c**) Mid-span comparsion of composite beams.

According to the deflection values measured at the bottom of the mid-span and the top of the plate end of the two specimens, the load-deflection curves of L1 and L2, were drawn, respectively, as shown in Figure 7a,b. The mid-span deflection of specimens L1 and L2 were compared, as shown in Figure 7c.

As can be seen from Figure 7a,b, in the loading process of two composite beam specimens, the end deflection deformation was small, and basically elastic. When the specimens were close to failure, the deflection change increased. The mid-span deflection of the two composite beams underwent obvious elastic, plastic and ductile failure stages. Compared with the test phenomenon, the micro-detachment expansion stage started from the initial and middle part of the elastic stage and completely covered the plastic stage. According to the load-deflection curve, the slope of the curve was basically unchanged during the initial loading process, and this stage was regarded as the elastic stage. As the load gradually increased, the slope of the curve decreased, and this stage was regarded as the plastic stage. With the further increase of the load to the ultimate bearing capacity, the deformation was maintained for a period of time without fracture. Compared with the test phenomenon, the micro-detachment appeared in the elastic stage, and the micro-detachment expansion stage started in the early part of the elastic stage and completely covered the plastic stage. Then the concrete body in the mid-span was crushed and the steel plate in the mid-span was buckled and deformed, and the composite beam was completely destroyed.

Figure 7c shows that when the load on the two composite beam specimens is less than 110 kN, the deformation curve is relatively flat, and the slope of the curve does not change significantly, indicating an elastic deformation in this stage, in which the angle of the internal steel plate has little effect on its elastic deformation. When the load on the composite beam L1 reaches 106 kN, the slope of the curve begins to decrease and it enters the plastic deformation. At this time, its mid-span deflection value is 8.84 mm, which slightly exceeds the limit state value for its normal service. Correspondingly, when the load on composite beam L2 reaches 103 kN, it enters the plastic deformation stage. At this time, its mid-span deflection value is 8.78 mm, which basically reaches the limit state value for normal service. After entering the plastic deformation stage, the outer steel plate on the side of the composite beam deformed greatly, which reduced the restraint on the internal concrete body, making the stiffness of the composite beam reduced and the deformation increased. As the load increases to 134 kN, the composite beam L1 reaches its ultimate bearing capacity; the composite beam L2 reaches its ultimate bearing capacity when a 123 kN force is loaded. It can be inferred that the ultimate bearing capacity of the composite beam would decrease as the angle of the porous internal steel plate increases.

### 4.3. Load-Strain Curve

#### 4.3.1. Load-Strain Curve of L1

**Figure 8:**
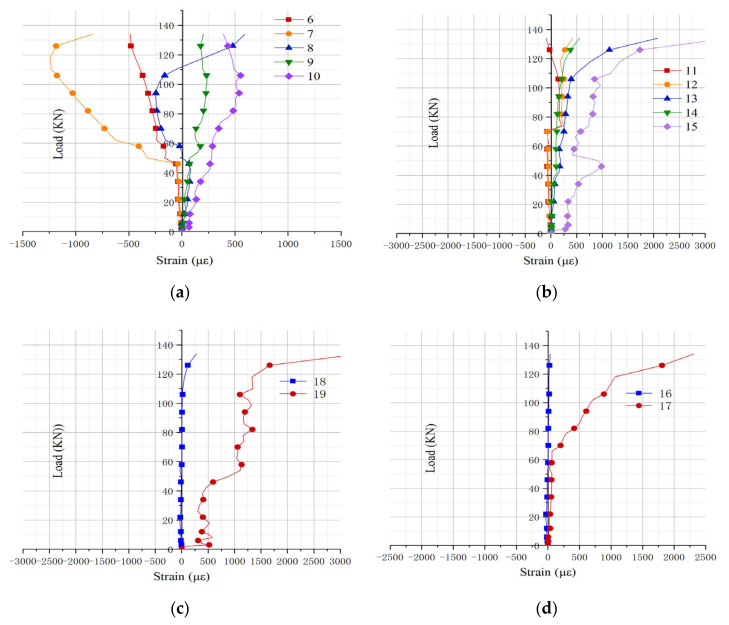
Load-strain curve of different measuring points of L1: (**a**) 6–10 strain gauge; (**b**) 11–15 strain gauge; (**c**) 18–19 strain gauge; (**d**) 16–17 strain gauge.

According to the strain value collected by the strain gauge, the load-strain curve [29,30] of the composite beam specimen L1 was drawn, as shown in Figure 8. Taking into account the symmetry of the measuring points, only the curve of the mid-span and one-side position was drawn.

It can be seen from Figure 8a that, since the strain values of measuring points 7 and 8 are close to zero, the tension-compression interface at the third point in the height direction of the composite beam specimen L1 is about 150 mm–200 mm in height. When the loading value reaches 50 kN, the strains at measurement points 6–9 change greatly. This is due to the slight detachment between the middle-span steel plate and the concrete body, which causes the bulge of steel plate and produces a sudden change in strain. This conclusion is consistent with the experimental test. Considering the bulge of the steel plate, the data collected at measuring points 7 and 8 are abandoned. It can be seen from Figure 8b that when the loading value reaches 74 kN, the strain values of measuring points 11 and 12 turn from negative to positive value. At this time, the tension zone and compression of the composite beam specimen L1 at the third point have shifted. From Figure 8a,b, it can be seen that before the loading reaches 106 kN, the slope of the curve remains basically unchanged, and the composite beam L1 is in the elastic deformation stage; when the load exceeds 106 kN, the slope of the curve keeps decreasing, and the composite beam L1 is in the plastic stage; when the load reaches 134 kN, composite beam L1 is crushed.

It can be seen from Figure 8c,d that on the bottom steel plate of the composite beam L1, the strain at the measuring points are all positive values, and the area where the bottom measuring points are located is generally in tension. Measuring point 16 and measuring point 18 are located near the connection between the inner porous steel plate and the outer steel plate. The local stiffness is relatively large while the deformation is small, and the composite beam remains in the elastic deformation stage under a load < 118 kN. The measuring point 17 is far away from the joint between the internal porous steel plate and the outer steel plate. The deformation there is large; when the load is smaller than 74 kN, the slope of the curve remains roughly unchanged, and it is in the stage of elastic deformation. Then, with the increase of load, it enters the plastic stage until it is crushed.

#### 4.3.2. Load-Strain Curve of L2

**Figure 9:**
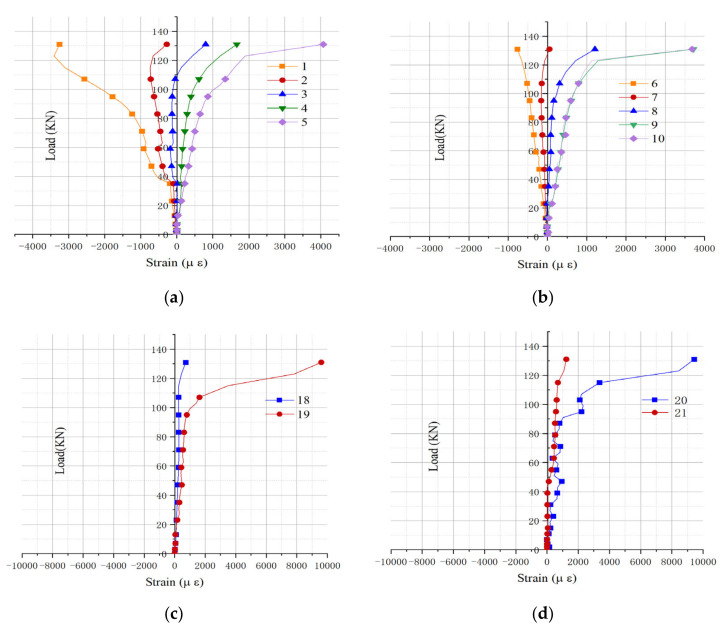
Load-strain curve of different measuring points of L2: (**a**) 1–5 strain gauge; (**b**) 6–10 strain gauge; (**c**) 18–19 strain gauge; (**d**) 20–21 strain gauge.

According to the strain value collected by the strain gauge, the load-strain curve of composite beam specimen L2 was drawn, as shown in Figure 9. Considering the symmetry of the measuring points, only the mid-span and one-side curves were drawn.

It can be seen from Figure 9a that since the strain values of measuring points 2 and 3 are close to zero, the tension-compression interface at the third point in the height direction of the composite beam specimen L2 is about 150 mm–200 mm in height. When the load reached 39 kN, the absolute value of the curve slope of the measuring points 1 and 2 suddenly increases and suddenly decreases when it is loaded to 63 kN. This is due to the extra deformation caused by the extrusion force when the force transmission bearing is against the steel plate. As can be seen from Figure 9b, the strain value of measuring point 7 that lies in the tension-compression interface at the third point is close to zero. Measuring points 8–10 are all under tension strain and the strain value is larger when it is farther away from measuring point 7. It can be seen that the tension zone has a smaller strain value when it is closer to the tension-compression interface. This is also the same for the compression zone. It can be seen from Figure 9a,b that before the load reaches 103 kN, the slope of the curve remains basically unchanged, and the composite beam L2 is in the elastic stage; when the load exceeds 103 kN, the slope of the curve decreases continuously, and the composite beam L2 is in plastic stage; when the load increases to 123 kN, composite beam L2 is crushed.

According to Figure 9c,d, on the bottom steel plate of composite beam L2, the measuring points are generally under tension and the deformation is relatively small. The measuring point 18 and the measuring point 21 are located close to the junction of the internal steel plate and the outer steel plate. Its local stiffness is relatively large, so its deformation is relatively small. Before being loaded to 123 kN, it is always in the elastic deformation stage, and some obvious deformation only occurs until the failure stage due to the excessive deformation of the composite beam specimen. The measuring point 19 and the measuring point 20 are located far away from the joint between the inner porous steel plate and the outer steel plate. The deformation constraint is small and the deformation is large. Before loading to 91 kN, the steel plate remains in the elastic deformation stage. The slope of the curve remains roughly unchanged. After that, as the load increases, the deformation becomes more obvious until the specimen L2 is crushed. At this time, the slope of the curve decreases greatly.

## 5. Finite Element Simulations

### 5.1. Model Simplification 

In the multi-cavity steel-concrete composite beam model, the function of the circular hole on the steel plate with internal holes is to provide the cohesive force between the concrete bodies in the cavities [31,32]. To simplify the calculation, the bond-slip effect between the concrete body and the steel plate or other concrete bodies is not taken into consideration, so the role played by the circular hole is replaced. Therefore, the internal porous steel plate is simplified as a steel plate, and its pores are converted into a porosity (the pore volume of steel plate used in the test/occupied volume of steel plate ≈50%). Then, the equivalent reduction is carried out on to the internal plate thickness.

### 5.2. Material Constitutive Model

#### 5.2.1. Steel Constitutive Relation

**Figure 10:**
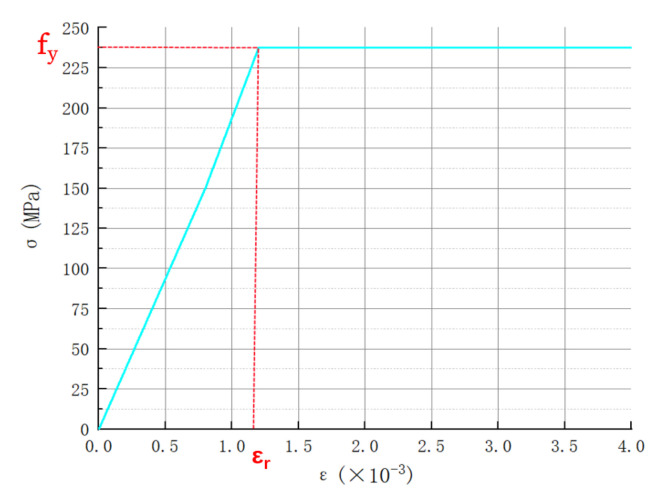
Constitutive relations of the steel sheet.

According to “Code for Design of Concrete Structures” (GB50010-2010) [26], combined with the setting of ANSYS, the constitutive relation of the steel selected in this paper is shown in Figure 10, which is an ideal elastic-plastic model, and its expression is shown in Equation (1).

(1) $$\left\{\begin{array}{c} \varepsilon \;\leq \;\varepsilon_{r},\;\sigma =\varepsilon_{s}E_{s}\\ \;\;\varepsilon >\varepsilon_{r},\;\sigma =\sigma_{s} \end{array}\right.$$

where:

$E_{s}$—the elastic modulus of steel;
$\varepsilon_{r}$—the yield strain of the steel, $\;\varepsilon_{r}=f_{y}/E_{s}$, $f_{y}$—the steel yield strength.

#### 5.2.2. Constitutive Relation of Concrete

**Figure 11:**
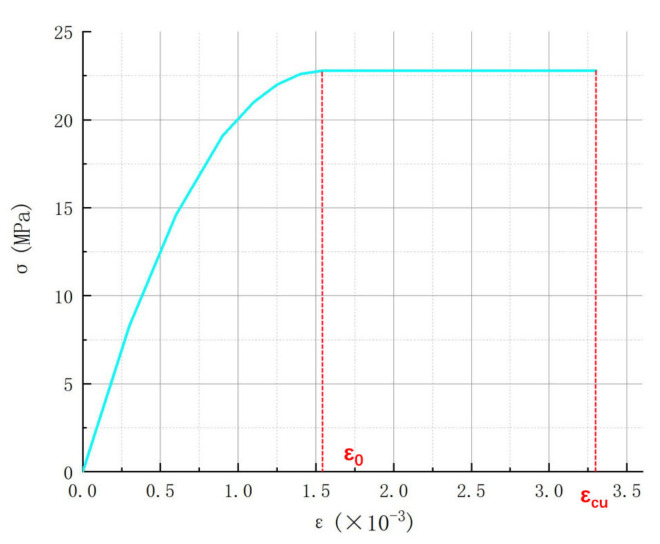
Constitutive relation of concrete.

According to “Code for Design of Concrete Structures” (GB50010-2010) [26], the concrete constitutive relation selected in this paper is shown in Figure 11, and its expression is shown in Equation (2).

(2) $$\left\{\begin{array}{c} Ascent:0\leq \varepsilon \leq \varepsilon_{0},\;\sigma =f_{c}\left[\frac{2\varepsilon }{\varepsilon_{0}}-{\left(\frac{\varepsilon }{\varepsilon_{0}}\right)}^{n}\right]\\ Descending\;segment:\varepsilon_{0}\leq \varepsilon \leq \varepsilon_{cu},\varepsilon =f_{c} \end{array}\right.$$

(3) $$n=1/60\left(f_{cu,k}-50\right)$$

where:

$f_{c}$—Design value of concrete axial compressive strength;
$\varepsilon_{0}$—peak strain, 0.002 for C30 concrete;
$\varepsilon_{cu}$—Ultimate strain, take 0.0033 for C30 concrete;
n—Coefficient, when the calculated *n* value is greater than 2.0, it is 2.0;
$f_{cu,k}$—Standard value of compressive strength of concrete cube.

#### 5.2.3. Model Parameters

According to the material properties, SHELL 181 element is selected for the multi-cavity steel plate frame in ANSYS, and SOLID 65 element is selected for the internal concrete body [33,34]. The concrete used in the model is simulated by an ideal homogeneous element, the strength grade is C30, the density is $2.50\times 10^{-6}$ kg/mm^3^, the elastic modulus is $3\times 10^{4}$ MPa, and Poisson’s ratio is 0.2; the steel strength grade is Q235, the density is $7.85\times 10^{-6}$ kg/mm^3^, the elastic modulus is $2\times 10^{5}$ MPa, and Poisson’s ratio is 0.3. The parameters of the template model of the multi-cavity steel-concrete composite beam are shown in Table 3.

### 5.3. Model Calculation

Surface loads and constraints are applied at the positions corresponding to the composite beam model and the test.

The constraints on the left are UX, UY, UZ, ROTX, and the constraints on the right are UY, UZ, which constitute the boundary conditions of the simply supported constraints. The force corresponding to the composite beam L1 at the elastic limit is 106 kN, and 103 kN for L2. Considering the initial load preload value of 4.7 kN on the composite beam specimen L1, the initial load preload value of 6.0 kN on specimen L2, and the self-weight of the upper beam transmitting load 7.7 kN, the load on the model LB-001 in the simulation needs to be increased by 12.4 kN, and the load on the model LB-002 needs to be increased by 13.7 kN.

To facilitate calculation and compare the relationship between the two models, the loads of the two models are taken as 120 kN for model ANSYS finite element [35,36,37] calculation.

### 5.4. Finite Element Simulation Results

**Figure 12:**
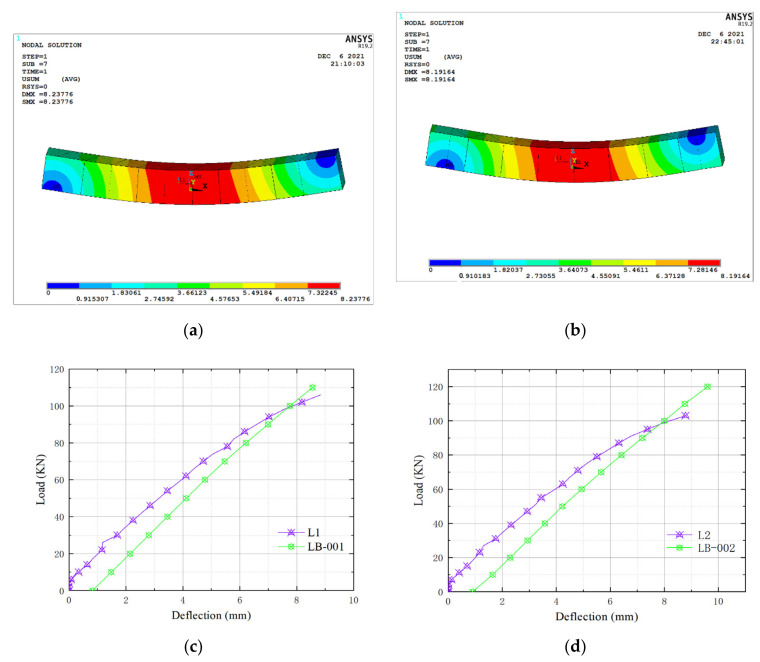
Comparison between software simulation results and test results: (**a**) Deflection cloud diagram of LB-001; (**b**) Deflection cloud diagram of LB-002; (**c**) Load-deflection comparison chart between L1 and LB-001; (**d**) Load-deflection comparison between of L2 and LB-002.

In the displacement cloud diagram made by ANSYS, DMX in the upper left corner represents the maximum displacement value. Under the action of the applied load, the model deflection diagrams of LB-001 and LB-002 are shown in Figure 12a,b. The maximum deflection values of the multi-cavity steel-concrete composite beam models LB-001 and LB-002 appear at the mid-span position and the deflection deformation at both ends is small, which are consistent with the test results.

When the two composite beams are loaded to 106 KN, L1 and L2 reach the normal service limit state. When loaded to 106 KN, the maximum mid-span deflection values of L1 and L2 are 8.837 mm and 8.780 mm, respectively. It can be seen from Figure 12c,d that the mid-span deflection values of LB-001 and LB-002 are 8.238 mm and 8.192 mm, respectively, which are smaller than the test results. Because the self-weight of the upper force transmission beam and the preloading value of the initial load are considered in the simulation, the initial deflection value of the model is not zero. Compared with LB-001 and LB-002, the load-deflection curves of L1 and L2 are in the elastic deformation stage at the initial stage. In the middle of loading, the slope of the two curves decreases, and both of them enter the plastic deformation stage. Moreover, the plastic deformation degree of models LB-001 and LB-002 is low, which can be regarded as linear change. Considering that there is no detachment between steel plate and concrete body in the simulation process of models LB-001 and LB-002, the plastic deformation degree is low, which is more consistent with the reality. The deviations of the mid-span deflection values of ANSYS simulation and the test are 6.78% and 6.70%, respectively. Therefore, the calculation results of the model are more accurate and reliable to a certain extent, which can be used for the subsequent analysis of influencing factors.

### 5.5. Analysis of Influencing Factors

#### 5.5.1. Finite Element Simulation Results

To comprehensively analyze the influence of various related factors on the mechanical properties of multi-cavity steel-concrete composite beams, four factors including concrete strength, steel strength, porosity, and angle are studied, and four different values are set for each influence factor. Based on the model with a steel plate angle of 60°, the factors are given different values, and thus their maximum deflection values under the same load and the different load values under the same deflection are obtained. The deflection is set as 8.8 mm because the deflection limit of normal service of this composite beam is $L_{0}/250=8.8\;\mathrm{mm}$. When the deflection reaches 8.8 mm, the corresponding load value is the ultimate load value for normal service.The composite beam parameters and simulation results are summarized in Table 4. 

According to Table 4, the analysis and simulation results show that:

(1) Under the same load, as the concrete strength increases, the mid-span deflection value of the composite beam model decreases. When it reaches the deflection limit, the bearing capacity is positively related to concrete strength. Concrete plays a compressive role in the whole composite beam. The higher its strength, smaller the stress deformation; the greater the overall stiffness, better the mechanical performance.
(2) Under the same load, as the strength of the steel increases, the mid-span deflection value of the composite beam model decreases. When the deflection limit is reached, the bearing capacity increases with the strength of the steel. The steel plate plays the role of tensile resistance and restraining the deformation of the concrete body in the overall composite beam. After reaching its yield strength, its influence on the model becomes more prominent.
(3) Under the same load, the mid-span deflection of the composite beam model increases with the increase of the porosity rate of steel plate. When the beam reaches the deflection limit, the bearing capacity decreases with the increase of the hole rate. The porous steel plate is the cross-section shear member in the composite beam. When its porosity increases, its volume decreases, the overall strength of the composite beam decreases, the shear resistance decreases, and the deformation increases.
(4) Under the same load, the mid-span deflection of the composite beam model increases when the angle of the internal steel plate increases. When it reaches the deflection limit, the bearing capacity decreases with the increase of the angle. According to simulation results, it is found that the impact of the porous steel plate on the bearing capacity is more obvious when the angle of the steel plate is relatively small or large. However, in the angle range from 60° to 75°, the corresponding impact is slight. The porous steel plates are the cross-section shear component in the composite beam. Its length is $L/\sin\frac{\alpha }{2}$. When the beam span is constant, the length of steel plate decreases with the increase of the angle, and thus the amount of steel used per unit volume inside the beam becomes small, which reduces the bearing capacity of the composite beam.

#### 5.5.2. Deflection-Load Curve of Influencing Factors

**Figure 13:**
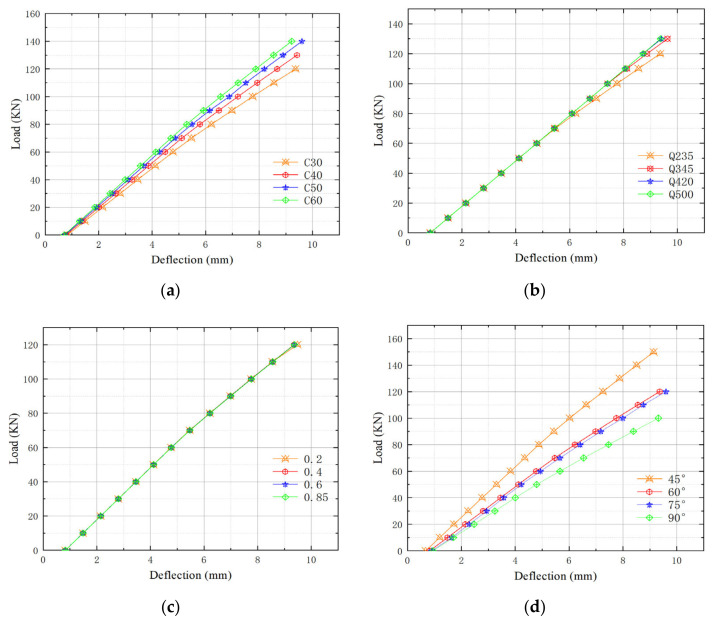
Deflection-load curve of multi-cavity steel-concrete composite beam: (**a**) Different concrete strength; (**b**) Different steel strength; (**c**) Different porosity rate; (**d**) Different angles of internal steel plate.

The simulation results of the loading process of the models are further summarized, and the corresponding deflection-load curves are present in Figure 13.

It can be seen from Figure 13a that with the increase of concrete strength, the slope of the curve increases and the deformation becomes slighter. In the four models, the load-deflection curves are all linearly at the initial stage. As the load increases, the deformation increases linearly. When the stress and deformation reach a certain level as the increase of the load, the plastic deformation of the concrete in the composite beam leads to the deformation of the steel plate, and then the composite beam enters the plastic deformation stage.

It can be seen from Figure 13b that as the strength of steels increase, the slope of the curve becomes larger and the deformation of the composite beam becomes smaller. When the steel plate stress in the composite beam model does not reach its yield strength, the influence of the steel strength is slight, and the load that reaches the same deflection value is the same. When the composite beams using the steel strength of Q235 and Q345 reach their yield strength, the steel yields, plastic deformation appears and the load-bearing capacity decreases. This phenomenon is more significant for composite beams using lower yield strength. When Q420 and Q500 steels are used, the maximum stress never exceeds the yield strength of the steel, so the deflection deformation of the model always increases linearly.

It can be seen from Figure 13c that a higher porosity of steel plate results in a lower slope of the curve and larger deformation of the model. The simulation result of the four models shows linear change in the initial stage of loading. Then the slope of the curves gradually decreases slightly, and small plastic deformation appears. The impact of porosity of steel plate on composite beam is not great. Changing the porosity makes little difference on the results.

It can be seen from Figure 13d that a greater angle of porous steel plate leads to a lower slope of the curve and greater deformation. The simulation result curves of the four models all showed linear changes in the initial stage of loading, and then the slope of the curve gradually decreases slightly. Plastic deformation takes place, but the deformation is not obvious. Observing the slope of the curve, it is found that with the increase of the angle of the steel plate, the slope change from 60° to 75° is smaller than the other two intervals.

## 6. Conclusions

In this paper, a new type of multi-cavity steel-concrete composite beam is proposed. Its mechanical properties and the influence of concrete strength, steel strength, cavity ratio and the angle of the internal steel plate on the mechanical properties of the composite beam are investigated by full-scale static load test and ANSYS finite element analysis at room temperature.

Based on the test and theoretical analysis, the following conclusions are drawn:

(1) During the loading process, before the multi-cavity steel-concrete composite beam reaches its normal service limit state, the deformation basically shows a linear change. As the increase of the load, the plastic deformation becomes obvious. In the failure stage, the middle-span steel plate is convex and the concrete are crushed, which can still provide a certain bearing capacity.
(2) By comparing the results obtained by ANSYS finite element modeling analysis with the test data, the credibility of the adopted modeling analysis method is confirmed, which can be used to carry out a further analysis. In the Ansys model, there is no small detachment between the steel plate and the concrete body in the mid-span position. Therefore, the plastic deformation and the overall deformation there are small before they reached the normal service limit state in the loading process, but this does not affect the use of this model for bearing capacity analysis of normal service.
(3) For the multi-cavity steel-concrete composite beam, with other factors being constant, the increase of concrete strength and steel strength can increase its bearing capacity, while the increase of internal steel plate porosity and angle will reduce its bearing capacity.
(4) Multi-cavity steel-concrete composite beams have large bearing capacity, good ductility and integrity.

## Figures and Tables

**Table 1:** Parameter of composite beam specimen.

Specimen	Cross Section (mm)	Span (mm)	Measured Size (mm) (*b* × *h* × *L* × *t*_1_ × *t*_2_)	*α* (°)	*r* (mm)	*d* (mm)	*L*_0_ (mm)	h/b
L1	200×300	2500	201.5×303.5×2510.0×1.0×1.7	60	10	30	2210.0	1.51
L2	200×300	2500	201.0×302.5×2509.5×1.0×1.7	75	10	30	2209.5	1.50

**Table 2:** Load segmented and hierarchical loading.

Specimen Number	Angle of Perforated Steel Plate	First Load (kN)			Second Load (kN)			Third Load (kN)			Fourth Load (kN)		
		Single-Stage	Initial Value	End Value	Single-Stage	Initial Value	End Value	Single-Stage	Initial Value	End Value	Single-Stage	Initial Value	End Value
L1	60°	1	0	6	2	6	14	4	14	110	8	110	142
L2	75°	1	0	5	2	5	15	4	15	107	8	107	139

**Table 3:** Model parameters of composite beams.

Serial Number	Size (mm$){}^{\circ}(b\times h\times L\times t_{1}\times t_{2}$)	α (°)	r (mm)	d (mm)	$L_{0}$ (mm)
LB-001	200 × 300 × 2500 × 1.0 × 1.7	60	10	30	2200
LB-002	200 × 300 × 2500 × 1.0 × 1.7	75	10	30	2200

**Table 4:** Summary of composite beam model parameters and results.

**Influencing Factors**	**Parameter Selection**	**Deflection under the Same Load**		**Load under the Same Deflection**	
		Load (kN)	Mid-Span Deflection (mm)	Mid-Span Deflection (mm)	Load (kN)
Concrete strength	C30(MPa)	106	8.238	8.798	113.03
	C40(MPa)	106	7.645	8.800	121.69
	C50(MPa)	106	7.234	8.800	128.60
	C60(MPa)	106	6.956	8.799	133.77
Steel strength	Q235(MPa)	106	8.238	8.798	113.03
	Q345(MPa)	106	7.838	8.799	119.01
	Q420(MPa)	106	7.803	8.797	121.14
	Q500(MPa)	106	7.803	8.804	121.26
Porosity	0.2(%)	106	8.230	8.797	113.12
	0.4(%)	106	8.235	8.800	113.08
	0.6(%)	106	8.240	8.800	113.01
	0.8(%)	106	8.246	8.799	112.93
Angle	45(°)	106	6.390	8.799	144.64
	60(°)	106	8.238	8.798	113.03
	75(°)	106	8.492	8.799	110.41
	90(°)	106	11.290	8.799	94.65

## Data Availability

The data used to support the findings of this study are available from the corresponding author upon request.
